# Supplementary material for: United States politicians’ tone became more negative with 2016 primary campaigns
Source: Sci Rep. 2023 Jun 28;13:10495. doi: 10.1038/s41598-023-36839-1 (PMC10307896; doi:10.1038/s41598-023-36839-1)
Supplement: Supplementary file 1 — Supplementary Information. [file 41598_2023_36839_MOESM1_ESM.pdf]

United States Politicians' Tone Became More Negative with 2016 Primary Campaigns

Supplementary Information

Contents

1 Figures **2**

2 Tables **10**

1 Figures

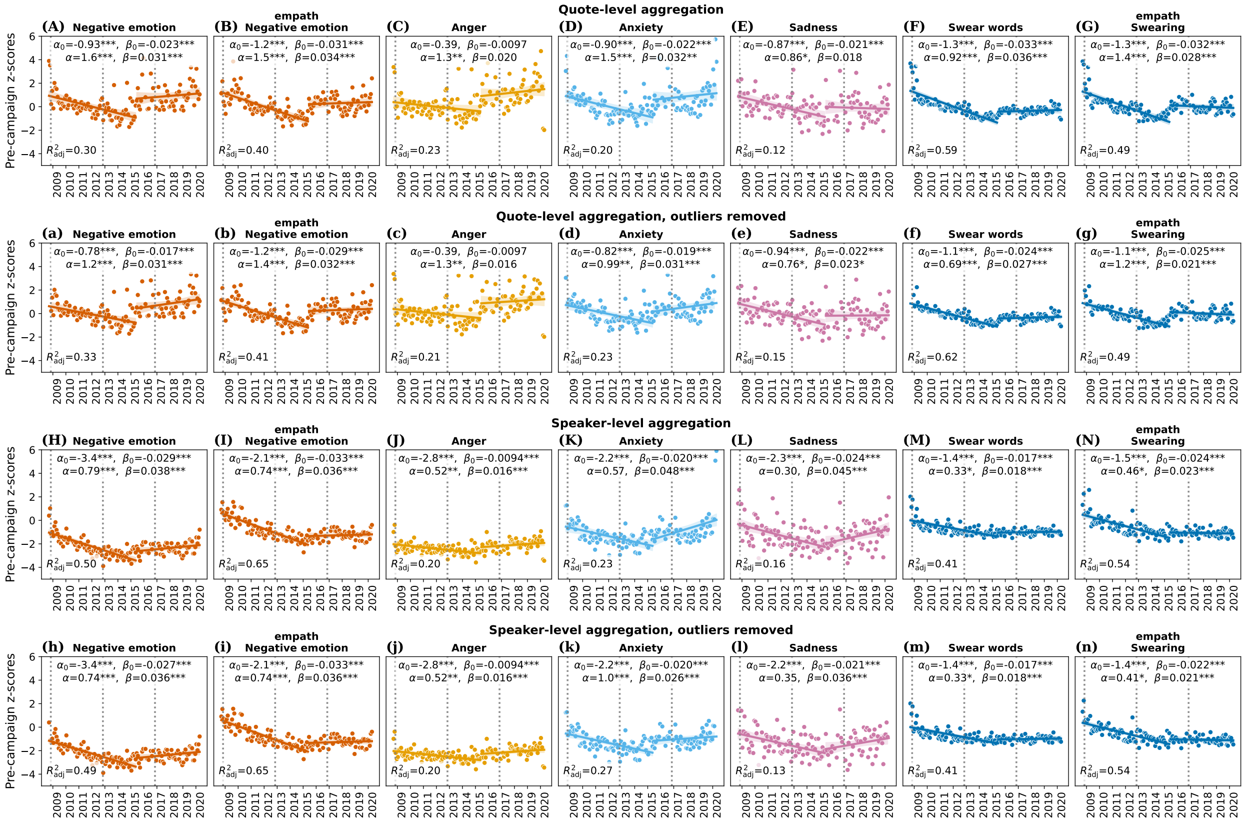

Figure 1: Quote-level and speaker-level aggregation, with and without outliers (+ empath control categories)

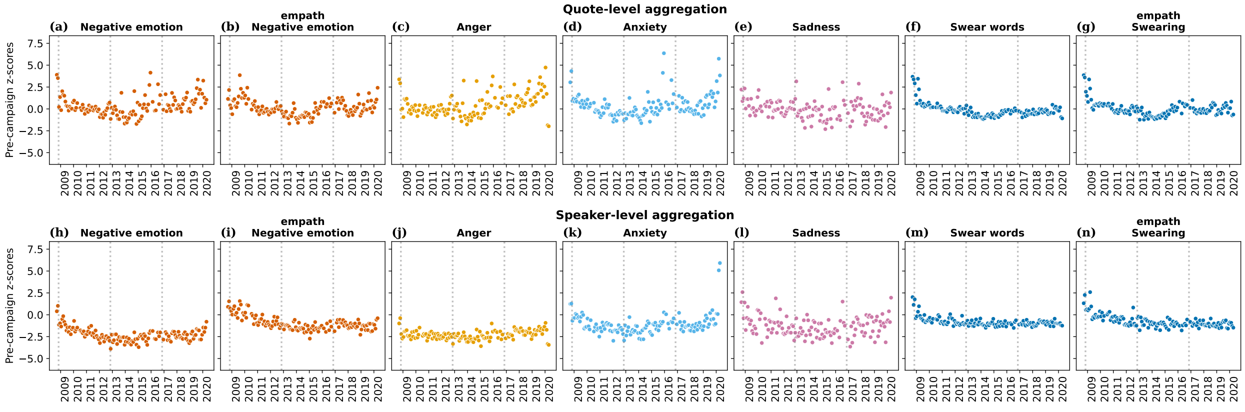

Figure 2: Standardized raw data for quote-level and speaker-level aggregates

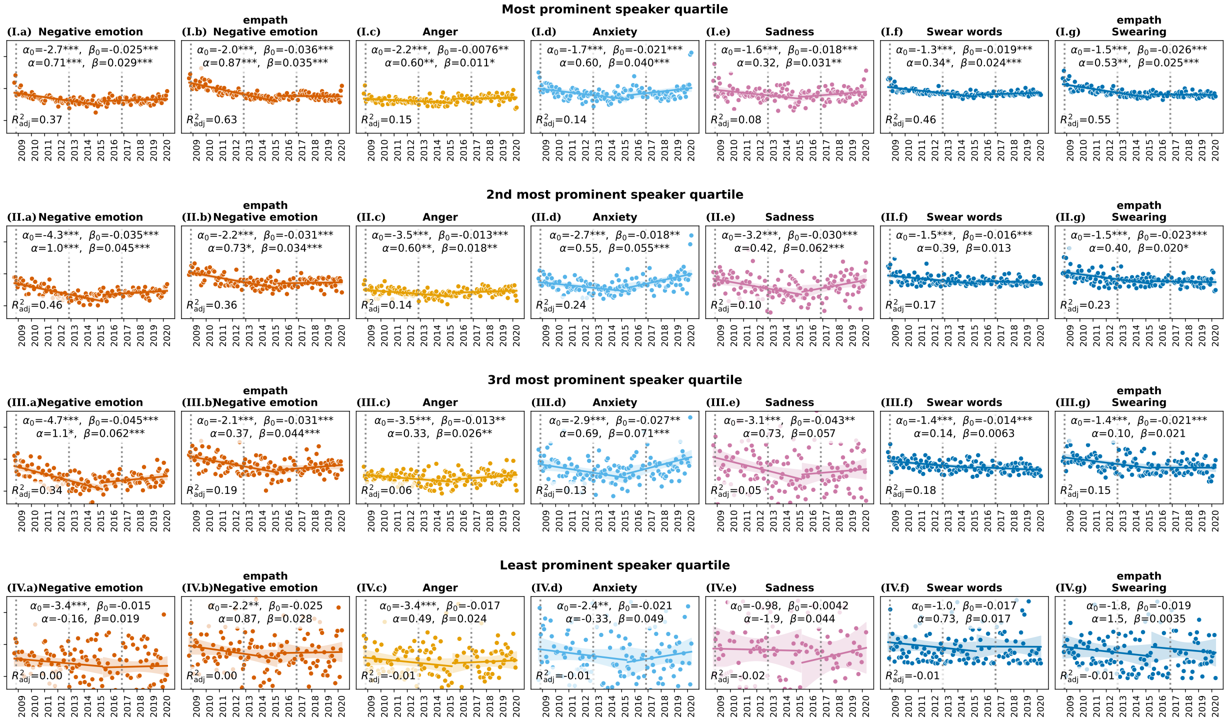

Figure 3: Speaker-level aggregation by prominence quartiles (extended version of Fig. 3 in the main text)

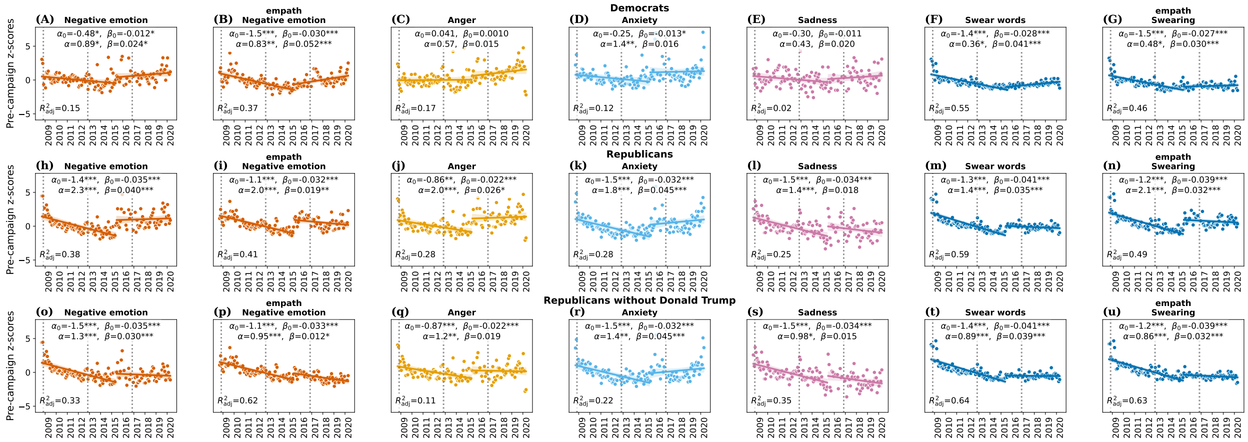

Figure 4: Quote-level aggregation split by party

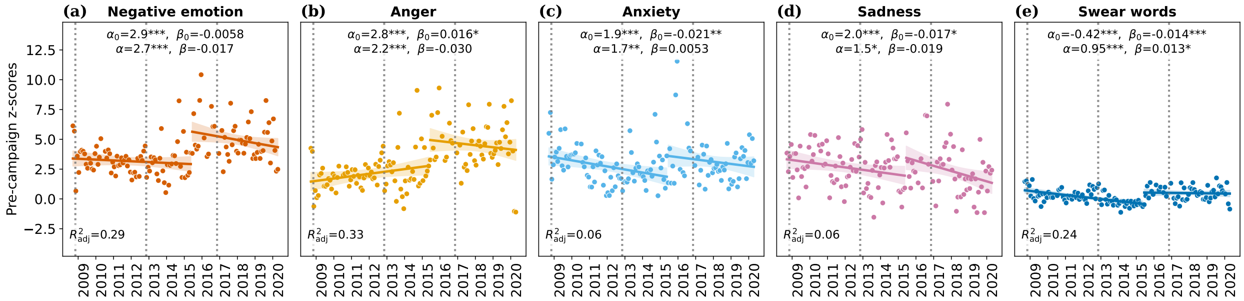

Figure 5: Quote-level aggregation limited to articles from the 65 most popular news websites according to YouGov (`https://today.yougov.com/ratings/entertainment/popularity/news-websites/all`)

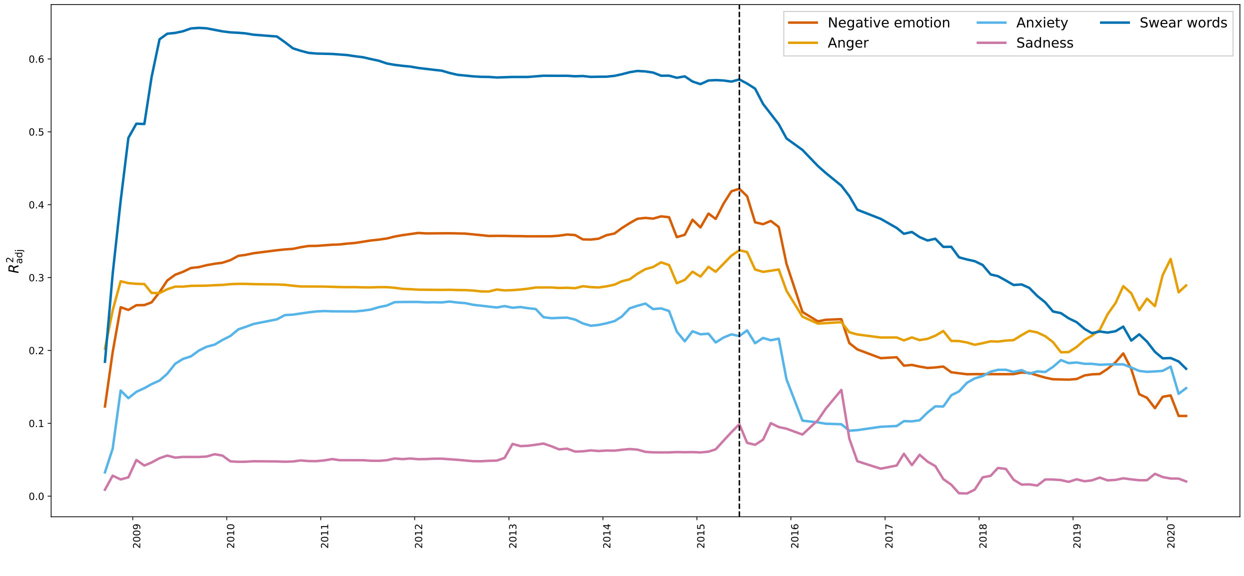

Figure 6: Adjusted R2 score of the OLS regression as a function of the discontinuity placement

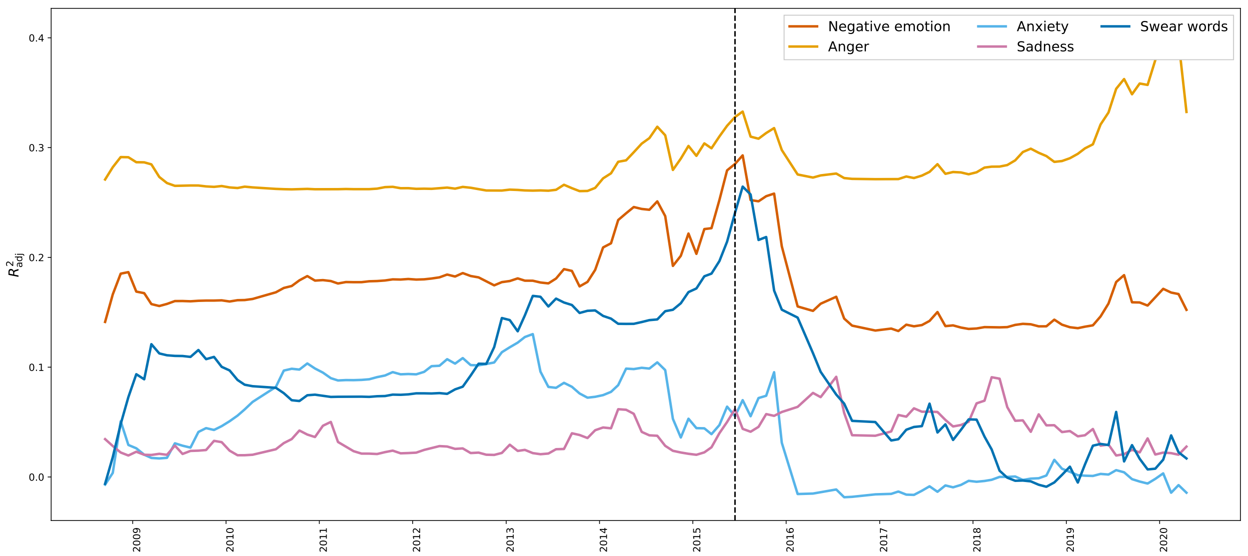

Figure 7: Adjusted R2 score of the OLS regression restricted to the 65 most popular news websites according to YouGov (`https://today.yougov.com/ratings/entertainment/popularity/news-websites/all`) as a function of the discontinuity placement

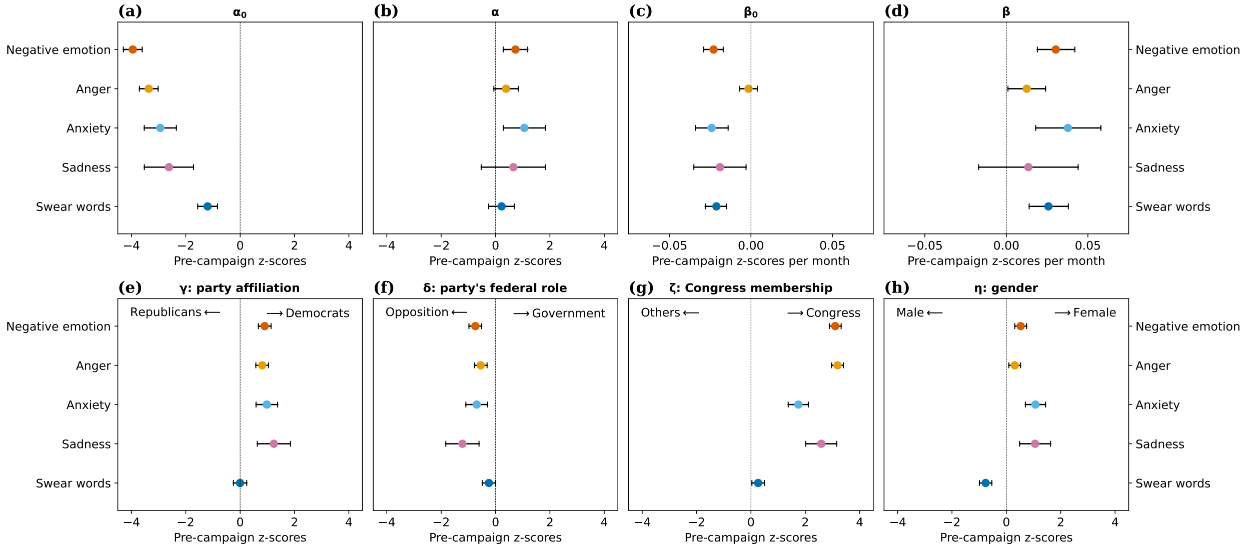

Figure 8: Biographic correlates of negative language based on speaker-level aggregation

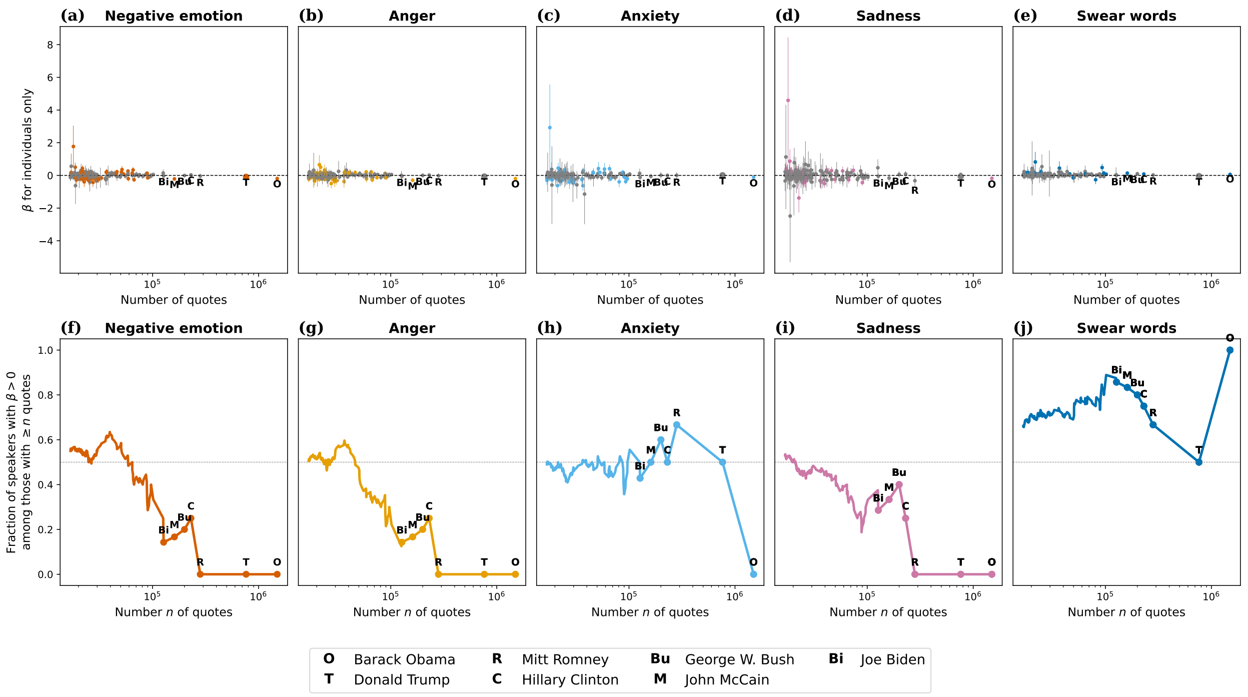

Figure 9: Regression parameter β resulting from an ordinary least squares regressions fitted separately to the time series of each of the 200 most quoted speakers. For detailed description of plot format, see caption of Fig. 5 in main text.

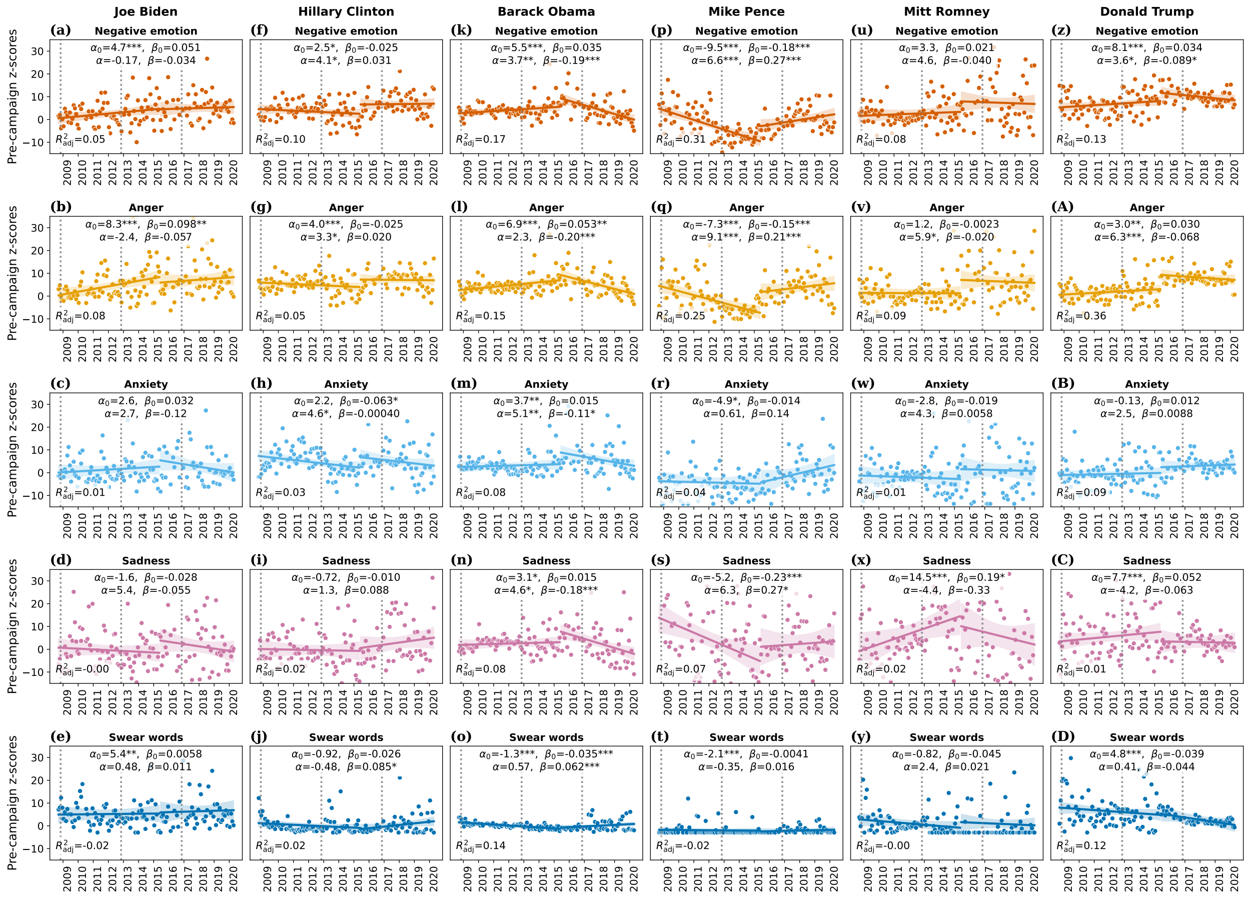

Figure 10: Quote-level aggregation for prominent politicians.

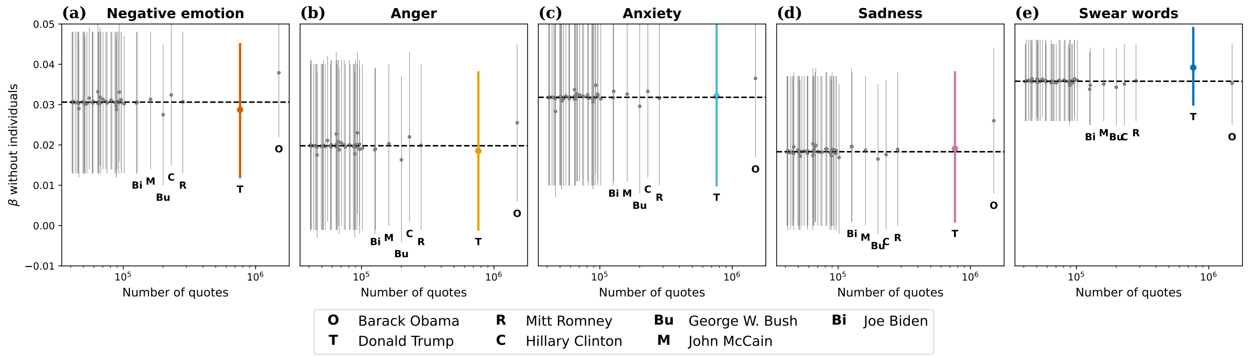

Figure 11: Regression parameter β obtained by removing all quotes by one target speaker, using the 50 most quoted speakers as target speakers

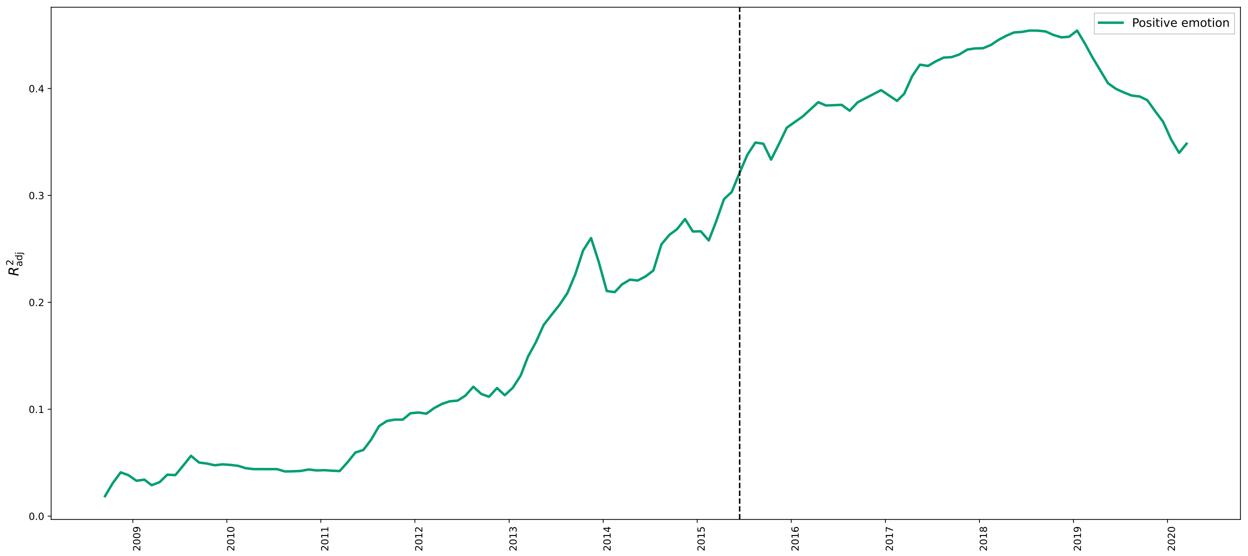

Figure 12: Adjusted R2 score of the OLS regression as a function of the discontinuity placement for positive emotions

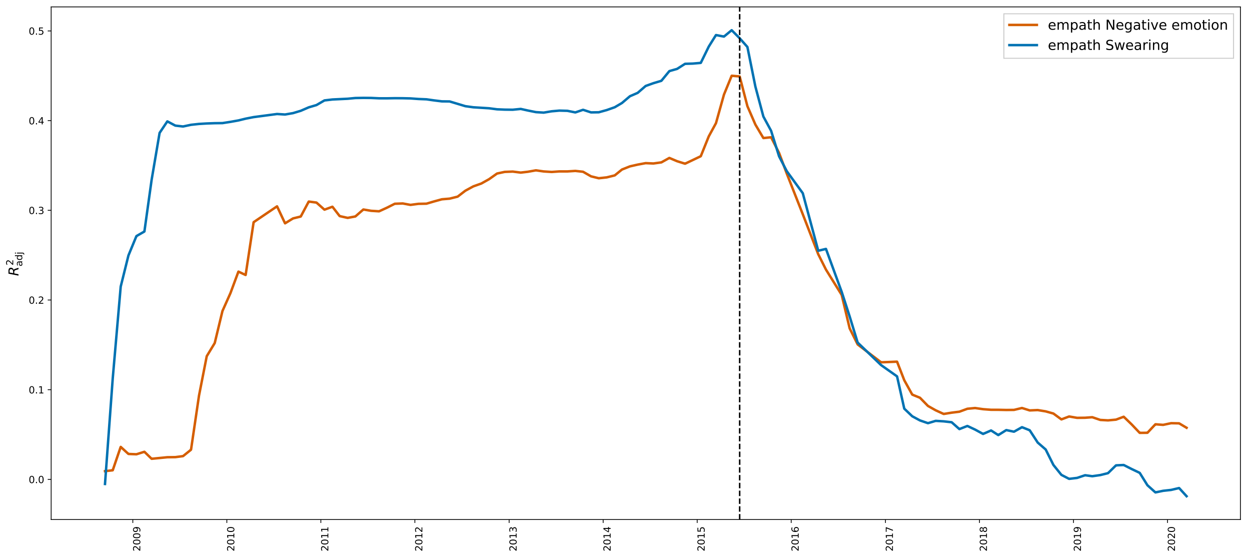

Figure 13: Adjusted R2 score of the OLS regression as a function of the discontinuity placement for empath-based scores

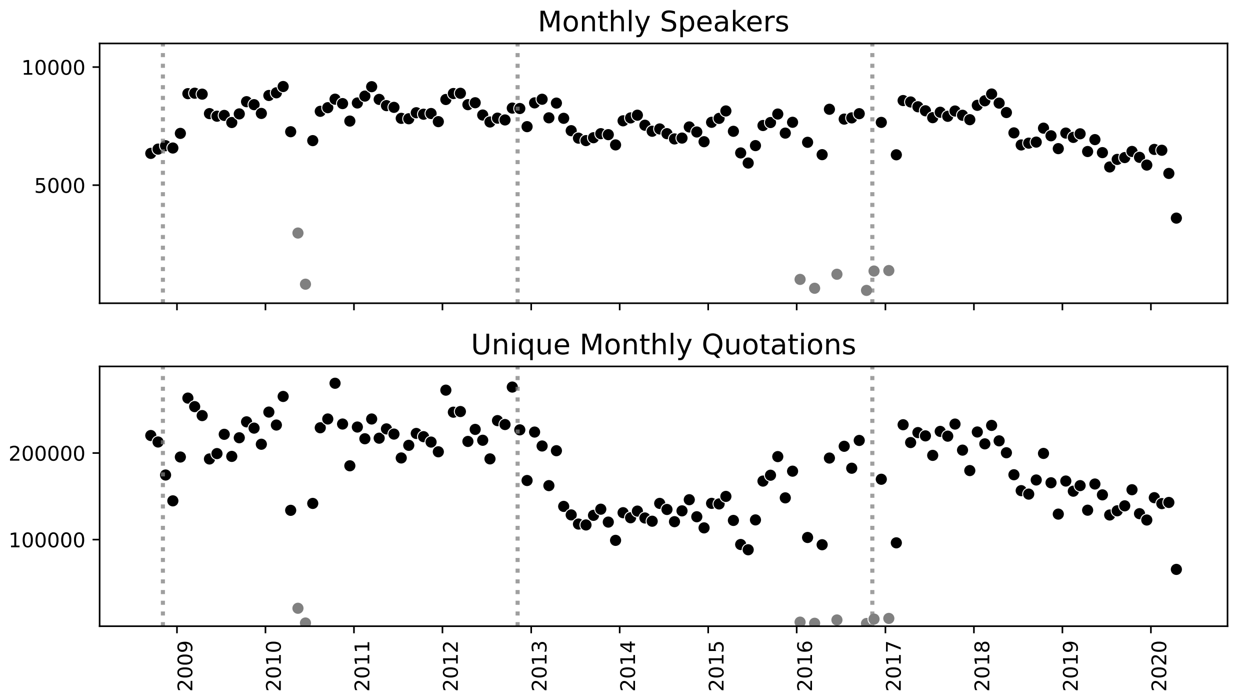

Figure 14: Monthly number of speakers and unique quotations in Quotebank (restricted to the 18,627 US politicians considered in the analysis). Months that were removed due missing data (cf. Materials and Methods in main text) are plotted in gray.

2 Tables

Table 1: **(Extended version of Table 1 in the main text) Key metrics for all word categories, including results for empath word categories**. Means μ and standard deviations σ were calculated over monthly quote-level aggregates from the pre-campaign period (September 2008 through May 2015). The value $n = 1/\mu$ in the fourth column implies that, in an average quote, on average every n-th word belongs to the respective category. The coefficients of variation, σ/μ, shown in the fifth column, allow to easily translate pre-campaign standard deviations (as shown on the y-axes of time series plots) into fractions of pre-campaign means. The most frequent words per category are listed in Supplementary Tables S2 and S3.

Word category	μ	σ	$1/\mu$	σ/μ
negative emotion	0.01853	0.00090	54.0	4.8%
empath negative emotion	0.00820	0.00054	122.0	6.6%
anger	0.00647	0.00055	154.6	8.4%
anxiety	0.00295	0.00022	339.4	7.3%
sadness	0.00351	0.00014	284.8	4.1%
swear words	0.00043	0.00015	2328.9	33.9%
empath swearing	0.00069	0.00013	1449.1	19.1%

Table 2: **The most common 30 words for each word category, before 15 June 2015**. Bold values deviate from the second period (after 15 June 2015) by more than one percentage point

Top N words	Negative emotion		Anger		Anxiety		Sadness		Swear words	
1	problem.*	4.35%	**fight.***	**7.67%**	risk.*	10.84%	low.*	10.88%	**hell**	**17.14%**
2	numb.*	3.20%	attack.*	4.98%	terror.*	8.35%	fail.*	9.85%	**damn.***	**8.68%**
3	fight.*	2.67%	threat.*	4.93%	worr.*	5.89%	lost	7.37%	heck	6.72%
4	low.*	2.11%	cut	4.87%	struggl.*	5.21%	hurt.*	5.22%	screw.*	6.62%
5	bad	1.92%	**war**	**4.83%**	pressur.*	5.16%	lose	5.11%	dumb.*	6.47%
6	fail.*	1.91%	defens.*	4.21%	doubt.*	4.43%	loss.*	4.23%	**dick**	**6.20%**
7	difficult.*	1.87%	kill.*	4.03%	avoid.*	4.17%	disappoint.*	4.12%	ass	4.95%
8	tough.*	1.79%	critical	3.71%	fear	3.52%	damag.*	3.88%	**shit.***	**4.86%**
9	wrong.*	1.77%	danger.*	3.10%	horr.*	2.50%	alone	2.89%	crap	3.41%
10	attack.*	1.73%	weapon.*	2.65%	overwhelm.*	2.47%	losing	2.61%	fuck	3.07%
11	risk.*	1.73%	argu.*	2.45%	vulnerab.*	2.38%	overwhelm.*	2.03%	fuckin.*	2.92%
12	threat.*	1.71%	victim.*	2.21%	afraid	2.34%	devastat.*	2.01%	butt	2.85%
13	cut	1.69%	violat.*	2.20%	craz.*	2.34%	defeat.*	1.96%	bitch.*	2.75%
14	war	1.68%	battl.*	2.01%	uncertain.*	2.18%	miss	1.91%	darn	2.68%
15	serious	1.51%	offens.*	1.99%	desperat.*	1.97%	traged.*	1.90%	bloody	2.28%
16	defens.*	1.46%	abuse.*	1.87%	stress.*	1.96%	reject.*	1.89%	piss.*	2.09%
17	lost	1.43%	aggress.*	1.68%	scare.*	1.87%	sad	1.81%	suck	2.04%
18	kill.*	1.40%	frustrat.*	1.53%	confus.*	1.76%	missed	1.61%	sucks	1.43%
19	terror.*	1.33%	destroy.*	1.42%	upset.*	1.75%	suffering	1.31%	sucked	1.22%
20	critical	1.29%	hate	1.23%	shame.*	1.71%	depress.*	1.30%	bastard.*	1.20%
21	danger.*	1.08%	blam.*	1.19%	distract.*	1.62%	missing	1.30%	butts	1.02%
22	hurt.*	1.01%	critici.*	1.06%	embarrass.*	1.61%	resign.*	1.23%	goddam.*	0.78%
23	lose	0.99%	fought	1.00%	guilt.*	1.33%	regret.*	1.23%	asshole.*	0.78%
24	worr.*	0.94%	murder.*	1.00%	emotional	1.27%	broke	1.21%	motherf.*	0.68%
25	weapon.*	0.92%	assault.*	0.98%	shake.*	1.21%	tragic.*	1.16%	nigger.*	0.62%
26	mistak.*	0.89%	outrag.*	0.92%	disturb.*	1.21%	abandon.*	1.06%	queer.*	0.55%
27	argu.*	0.85%	violent.*	0.88%	anxi.*	1.13%	isolat.*	1.00%	boob.*	0.49%
28	struggl.*	0.83%	hell	0.87%	alarm.*	1.04%	empt.*	0.95%	fucked.*	0.48%
29	pressur.*	0.82%	punish.*	0.85%	nervous.*	1.02%	suffer	0.93%	dang	0.47%
30	loss.*	0.82%	offend.*	0.83%	suspicio.*	0.92%	grave.*	0.93%	prick.*	0.47%
Total nr Expressions	499		184		91		101		53	
Total Matches	5763501		2005132		918339		1117453		101773	

Table 3: **The most common 30 words for each word category, after 15 June 2015**. Bold values deviate from the first period (before 15 June 2015) by more than one percentage point

Top N words	Negative emotion		Anger		Anxiety		Sadness		Swear words	
1	problem.*	3.79%	**fight.***	**9.15%**	risk.*	10.82%	low.*	11.05%	**hell**	**24.09%**
2	fight.*	3.36%	threat.*	5.72%	terror.*	8.92%	fail.*	9.05%	**damn.***	**11.48%**
3	numb.*	3.03%	attack.*	5.43%	worr.*	5.50%	lost	6.89%	dumb.*	7.11%
4	threat.*	2.10%	critical	3.90%	pressur.*	4.94%	hurt.*	5.38%	screw.*	6.71%
5	low.*	2.04%	**danger.***	**3.80%**	horr.*	4.27%	lose	4.95%	heck	6.08%
6	attack.*	1.99%	kill.*	3.60%	struggl.*	4.26%	damag.*	4.22%	**shit.***	**4.71%**
7	bad	1.95%	war	3.59%	fear	4.03%	loss.*	3.78%	ass	4.47%
8	risk.*	1.78%	defens.*	2.94%	doubt.*	3.91%	disappoint.*	3.73%	**crap**	**3.43%**
9	wrong.*	1.78%	weapon.*	2.82%	vulnerab.*	3.42%	alone	2.90%	darn	2.75%
10	fail.*	1.67%	cut	2.79%	avoid.*	3.20%	devastat.*	2.57%	fuckin.*	2.51%
11	terror.*	1.47%	victim.*	2.74%	craz.*	2.54%	traged.*	2.34%	bitch.*	2.47%
12	critical	1.43%	abuse.*	2.41%	overwhelm.*	2.38%	losing	2.32%	piss.*	2.42%
13	difficult.*	1.43%	violat.*	2.26%	afraid	2.34%	defeat.*	2.31%	butt	2.37%
14	tough.*	1.43%	hate	1.94%	shame.*	1.95%	sad	2.19%	suck	2.18%
15	danger.*	1.40%	argu.*	1.87%	shy.*	1.87%	overwhelm.*	2.12%	bloody	2.17%
16	serious	1.38%	offens.*	1.86%	uncertain.*	1.85%	reject.*	2.00%	sucks	1.70%
17	kill.*	1.32%	destroy.*	1.54%	desperat.*	1.79%	resign.*	1.66%	fuck	1.62%
18	war	1.32%	battl.*	1.53%	stress.*	1.77%	suffering	1.60%	sucked	1.39%
19	lost	1.28%	aggress.*	1.52%	confus.*	1.65%	miss	1.59%	motherf.*	1.09%
20	defens.*	1.08%	assault.*	1.44%	embarrass.*	1.56%	missed	1.54%	queer.*	0.95%
21	weapon.*	1.04%	frustrat.*	1.43%	disturb.*	1.55%	tragic.*	1.47%	butts	0.80%
22	cut	1.02%	murder.*	1.27%	scare.*	1.53%	missing	1.29%	bastard.*	0.78%
23	victim.*	1.01%	fought	1.08%	distract.*	1.51%	regret.*	1.15%	goddam.*	0.73%
24	hurt.*	1.00%	violent.*	1.06%	guilt.*	1.50%	isolat.*	1.13%	pussy.*	0.58%
25	lose	0.92%	blam.*	0.99%	upset.*	1.48%	abandon.*	1.11%	tit	0.53%
26	worr.*	0.91%	hell	0.95%	alarm.*	1.29%	broke	1.04%	asshole.*	0.52%
27	abuse.*	0.89%	critici.*	0.93%	emotional	1.22%	grave.*	1.01%	asses	0.49%
28	violat.*	0.83%	outrag.*	0.92%	anxi.*	1.17%	suffer	0.92%	dick	0.47%
29	mistak.*	0.82%	harass.*	0.92%	shake.*	0.95%	suffered	0.87%	dang	0.44%
30	pressur.*	0.81%	punish.*	0.79%	tension.*	0.80%	empt.*	0.83%	crappy	0.42%
Total nr Expressions	499		184		91		101		53	
Total Matches	3695279		1357629		608580		683988		53284	

Table 4: **OLS regression parameters for quote-level aggregation shown in Figure 2 in the main text**. SEs of coefficients are in parantheses. ***$p < 0.001$, **$p < 0.01$ and *$p < 0.05$

	negative emotion	anger	anxiety	sadness	swear words
α_0	-0.932***(0.209)	-0.391 (0.249)	-0.903***(0.263)	-0.867***(0.226)	-1.347***(0.117)
α	1.622***(0.337)	1.299** (0.402)	1.501***(0.424)	0.855* (0.365)	0.923***(0.189)
β_0	-0.023***(0.004)	-0.010 (0.005)	-0.022***(0.006)	-0.021***(0.005)	-0.033***(0.002)
β	0.031***(0.009)	0.020 (0.010)	0.032** (0.011)	0.018 (0.009)	0.036***(0.005)
R^2	0.315	0.251	0.215	0.136	0.599
Adj. R^2	0.299	0.234	0.197	0.116	0.590
No. Observations	132	132	132	132	132

Table 5: **OLS regression parameters for quote-level aggregation, excluding outliers**. SEs of coefficients are in parantheses. ***$p < 0.001$, **$p < 0.01$ and *$p < 0.05$

	negative emotion	anger	anxiety	sadness	swear words
α_0	-0.780***(0.184)	-0.391 (0.231)	-0.816***(0.187)	-0.944***(0.209)	-1.110***(0.075)
α	1.222***(0.299)	1.260** (0.382)	0.989** (0.317)	0.756* (0.340)	0.686***(0.120)
β_0	-0.017***(0.004)	-0.010 (0.005)	-0.019***(0.004)	-0.022***(0.004)	-0.024***(0.002)
β	0.031***(0.008)	0.016 (0.010)	0.031***(0.008)	0.023* (0.009)	0.027***(0.003)
R^2	0.347	0.224	0.250	0.168	0.632
Adj. R^2	0.331	0.206	0.232	0.149	0.623
No. Observations	129	129	127	130	128

Table 6: **OLS regression parameters for speaker-level aggregation shown in Figure 2 in the main text**. SEs of coefficients are in parantheses. ***$p < 0.001$, **$p < 0.01$ and *$p < 0.05$

	negative emotion	anger	anxiety	sadness	swear words
α_0	-3.413***(0.121)	-2.800***(0.105)	-2.170***(0.223)	-2.268***(0.238)	-1.374***(0.092)
α	0.790***(0.195)	0.522** (0.169)	0.573 (0.359)	0.304 (0.384)	0.328* (0.149)
β_0	-0.029***(0.003)	-0.009***(0.002)	-0.020***(0.005)	-0.024***(0.005)	-0.017***(0.002)
β	0.038***(0.005)	0.016***(0.004)	0.048***(0.009)	0.045***(0.010)	0.018***(0.004)
R^2	0.509	0.220	0.251	0.181	0.421
Adj. R^2	0.498	0.202	0.234	0.161	0.408
No. Observations	132	132	132	132	132

Table 7: **OLS regression parameters for speaker-level aggregation, excluding outliers**. SEs of coefficients are in parantheses. ***$p < 0.001$, **$p < 0.01$ and *$p < 0.05$

	negative emotion	anger	anxiety	sadness	swear words
α_0	-3.360***(0.115)	-2.800***(0.105)	-2.170***(0.150)	-2.206***(0.219)	-1.374***(0.092)
α	0.737***(0.184)	0.522** (0.169)	1.010***(0.245)	0.346 (0.354)	0.328* (0.149)
β_0	-0.027***(0.002)	-0.009***(0.002)	-0.020***(0.003)	-0.021***(0.005)	-0.017***(0.002)
β	0.036***(0.005)	0.016***(0.004)	0.026***(0.007)	0.036***(0.009)	0.018***(0.004)
R^2	0.498	0.220	0.286	0.153	0.421
Adj. R^2	0.486	0.202	0.269	0.132	0.408
No. Observations	131	132	130	129	132

Table 8: **OLS regression parameters for quote-level aggregation for Democrats shown in Figure 2 in the main text**. SEs of coefficients are in parantheses. ***$p < 0.001$, **$p < 0.01$ and *$p < 0.05$

	negative emotion	anger	anxiety	sadness	swear words
α_0	-0.481* (0.223)	0.041 (0.269)	-0.247 (0.296)	-0.297 (0.289)	-1.397***(0.110)
α	0.892* (0.360)	0.573 (0.433)	1.375** (0.477)	0.426 (0.466)	0.362* (0.178)
β_0	-0.012* (0.005)	0.001 (0.006)	-0.013* (0.006)	-0.011 (0.006)	-0.028***(0.002)
β	0.024* (0.009)	0.015 (0.011)	0.016 (0.012)	0.020 (0.012)	0.041***(0.005)
R^2	0.172	0.190	0.141	0.039	0.561
Adj. R^2	0.153	0.171	0.120	0.016	0.550
No. Observations	132	132	132	132	132

Table 9: **OLS regression parameters for quote-level aggregation for Republicans shown in Figure 2 in the main text**. SEs of coefficients are in parantheses. ***$p < 0.001$, **$p < 0.01$ and *$p < 0.05$

	negative emotion	anger	anxiety	sadness	swear words
α_0	-1.417***(0.231)	-0.856** (0.256)	-1.543***(0.259)	-1.469***(0.255)	-1.345***(0.143)
α	2.323***(0.372)	1.990***(0.413)	1.772***(0.418)	1.437***(0.411)	1.394***(0.231)
β_0	-0.035***(0.005)	-0.022***(0.005)	-0.032***(0.006)	-0.034***(0.005)	-0.041***(0.003)
β	0.040***(0.010)	0.026* (0.011)	0.045***(0.011)	0.018 (0.011)	0.035***(0.006)
R^2	0.394	0.299	0.295	0.267	0.601
Adj. R^2	0.380	0.282	0.278	0.249	0.592
No. Observations	132	132	132	132	132

Table 10: **OLS regression parameters for quote-level aggregation for Republicans, but excluding Donald Trump**. SEs of coefficients are in parantheses. ***$p < 0.001$, **$p < 0.01$ and *$p < 0.05$

	negative emotion	anger	anxiety	sadness	swear words
α_0	-1.456***(0.211)	-0.870***(0.237)	-1.548***(0.266)	-1.508***(0.244)	-1.369***(0.141)
α	1.252***(0.340)	1.156** (0.382)	1.414** (0.429)	0.979* (0.393)	0.889***(0.227)
β_0	-0.035***(0.005)	-0.022***(0.005)	-0.032***(0.006)	-0.034***(0.005)	-0.041***(0.003)
β	0.030***(0.009)	0.019 (0.010)	0.045***(0.011)	0.015 (0.010)	0.039***(0.006)
R^2	0.343	0.134	0.235	0.370	0.644
Adj. R^2	0.328	0.113	0.217	0.355	0.636
No. Observations	132	132	132	132	132

Table 11: **OLS regression parameters for aggregation on the most prominent speaker quartile**. SEs of coefficients are in parantheses. ***$p < 0.001$, **$p < 0.01$ and *$p < 0.05$

	negative emotion	anger	anxiety	sadness	swear words
α_0	-2.661***(0.131)	-2.217***(0.121)	-1.673***(0.243)	-1.611***(0.244)	-1.288***(0.089)
α	0.712***(0.210)	0.601** (0.194)	0.602 (0.391)	0.323 (0.394)	0.337* (0.143)
β_0	-0.025***(0.003)	-0.008** (0.003)	-0.021***(0.005)	-0.018***(0.005)	-0.019***(0.002)
β	0.029***(0.005)	0.011* (0.005)	0.040***(0.010)	0.031** (0.010)	0.024***(0.004)
R^2	0.387	0.170	0.162	0.099	0.468
Adj. R^2	0.373	0.150	0.143	0.078	0.456
No. Observations	132	132	132	132	132

Table 12: **OLS regression parameters for aggregation on the 2nd most prominent speaker quartile**. SEs of coefficients are in parantheses. ***$p < 0.001$, **$p < 0.01$ and *$p < 0.05$

	negative emotion	anger	anxiety	sadness	swear words
α_0	-4.272***(0.153)	-3.539***(0.141)	-2.721***(0.273)	-3.229***(0.398)	-1.534***(0.155)
α	1.012***(0.247)	0.599** (0.227)	0.547 (0.440)	0.417 (0.641)	0.391 (0.250)
β_0	-0.035***(0.003)	-0.013***(0.003)	-0.018** (0.006)	-0.030***(0.009)	-0.016***(0.003)
β	0.045***(0.006)	0.018** (0.006)	0.055***(0.011)	0.062***(0.017)	0.013 (0.006)
R^2	0.476	0.157	0.255	0.123	0.194
Adj. R^2	0.464	0.137	0.238	0.102	0.175
No. Observations	132	132	132	132	132

Table 13: **OLS regression parameters for aggregation on the 3rd most prominent speaker quartile**. SEs of coefficients are in parantheses. ***$p < 0.001$, **$p < 0.01$ and *$p < 0.05$

	negative emotion	anger	anxiety	sadness	swear words
α_0	-4.703***(0.260)	-3.539***(0.225)	-2.937***(0.455)	-3.128***(0.705)	-1.397***(0.186)
α	1.067* (0.419)	0.331 (0.363)	0.686 (0.733)	0.734 (1.136)	0.145 (0.300)
β_0	-0.045***(0.006)	-0.013** (0.005)	-0.027** (0.010)	-0.043** (0.015)	-0.014***(0.004)
β	0.062***(0.011)	0.026** (0.009)	0.071***(0.019)	0.057 (0.029)	0.006 (0.008)
R^2	0.352	0.081	0.149	0.068	0.198
Adj. R^2	0.337	0.060	0.129	0.046	0.179
No. Observations	132	132	132	132	132

Table 14: **OLS regression parameters for aggregation on the least prominent speaker quartile**. SEs of coefficients are in parantheses. ***$p < 0.001$, **$p < 0.01$ and *$p < 0.05$

	negative emotion	anger	anxiety	sadness	swear words
α_0	-3.425***(0.623)	-3.402***(0.600)	-2.429** (0.929)	-0.980 (1.738)	-1.042 (0.735)
α	-0.163 (1.005)	0.493 (0.967)	-0.327 (1.497)	-1.859 (2.801)	0.729 (1.185)
β_0	-0.015 (0.013)	-0.017 (0.013)	-0.021 (0.020)	-0.004 (0.037)	-0.017 (0.016)
β	0.019 (0.026)	0.024 (0.025)	0.049 (0.039)	0.044 (0.073)	0.017 (0.031)
R^2	0.023	0.015	0.015	0.006	0.009
Adj. R^2	0.000	-0.008	-0.008	-0.017	-0.014
No. Observations	132	132	132	132	132

Table 15: **OLS regression parameters for quote-level aggregation on speaker groups based on party affiliation, party's federal role, Congress membership, and gender**. SEs of coefficients are in parantheses. ***$p < 0.001$, **$p < 0.01$ and *$p < 0.05$

	negative emotion	anger	anxiety	sadness	swear words
α_0	-1.787***(0.183)	-1.520***(0.207)	-1.681***(0.203)	-1.198***(0.266)	-0.853***(0.100)
α	1.293***(0.240)	0.800** (0.271)	1.960***(0.265)	0.983** (0.347)	0.522***(0.131)
β_0	-0.013***(0.003)	0.002 (0.004)	-0.018***(0.004)	-0.013** (0.005)	-0.033***(0.002)
β	0.014* (0.006)	0.009 (0.007)	0.006 (0.007)	-0.006 (0.009)	0.043***(0.003)
γ: party affiliation	0.479***(0.124)	0.559***(0.140)	0.964***(0.137)	0.668***(0.179)	-0.193** (0.068)
η: gender	-0.215 (0.115)	-0.172 (0.130)	0.480***(0.128)	0.413* (0.167)	-0.828***(0.063)
ζ: Congress membership	3.412***(0.115)	3.767***(0.130)	1.734***(0.128)	2.255***(0.167)	-0.150* (0.063)
δ: party's federal role	-0.335** (0.124)	-0.185 (0.140)	-0.357** (0.137)	-0.976***(0.179)	-0.328***(0.068)
R^2	0.478	0.473	0.235	0.184	0.390
Adj. R^2	0.475	0.470	0.230	0.178	0.386
No. Observations	1056	1056	1056	1056	1056

Table 16: **OLS regression parameters for speaker-level aggregation on speaker groups based on party affiliation, party's federal role, Congress membership, and gender**. SEs of coefficients are in parantheses. ***$p < 0.001$, **$p < 0.01$ and *$p < 0.05$

	negative emotion	anger	anxiety	sadness	swear words
α_0	-3.949***(0.176)	-3.361***(0.175)	-2.938***(0.301)	-2.619***(0.462)	-1.196***(0.185)
α	0.734** (0.230)	0.388 (0.229)	1.058** (0.393)	0.658 (0.603)	0.223 (0.242)
β_0	-0.023***(0.003)	-0.002 (0.003)	-0.024***(0.005)	-0.019* (0.008)	-0.021***(0.003)
β	0.030***(0.006)	0.013* (0.006)	0.038***(0.010)	0.013 (0.016)	0.026***(0.006)
γ: party affiliation	0.904***(0.119)	0.810***(0.118)	0.983***(0.203)	1.242***(0.312)	-0.002 (0.125)
η: gender	0.528***(0.111)	0.308** (0.110)	1.070***(0.189)	1.057***(0.290)	-0.761***(0.116)
ζ: Congress membership	3.101***(0.111)	3.184***(0.110)	1.742***(0.189)	2.586***(0.290)	0.264* (0.116)
δ: party's federal role	-0.744***(0.119)	-0.544***(0.118)	-0.694***(0.203)	-1.219***(0.312)	-0.243 (0.125)
R^2	0.472	0.472	0.142	0.104	0.099
Adj. R^2	0.469	0.469	0.137	0.098	0.093
No. Observations	1056	1056	1056	1056	1056

Table 17: **Most frequently quoted politicians and the Wikidata identifiers (QID)**.

	QID	Name	Number of quotes	Party	Gender
1	Q76	Barack Obama	1499080	Democrat	M
2	Q22686	Donald Trump	762822	Republican	M
3	Q4496	Mitt Romney	281774	Republican	M
4	Q6294	Hillary Clinton	230306	Democrat	F
5	Q207	George W. Bush	200013	Republican	M
6	Q10390	John McCain	160805	Republican	M
7	Q6279	Joe Biden	127182	Democrat	M
8	Q43144	Sarah Palin	125980	Republican	F
9	Q170581	Nancy Pelosi	101583	Democrat	F
10	Q359442	Bernie Sanders	96701	Democrat	M
11	Q63879	Chris Christie	96361	Republican	M
12	Q22316	John Kerry	93036	Democrat	M
13	Q203966	Paul Ryan	92407	Republican	M
14	Q215057	Rick Perry	89975	Republican	M
15	Q23505	George H. W. Bush	88859	Republican	M
16	Q11673	Andrew Cuomo	88433	Democrat	M
17	Q1124	Bill Clinton	88173	Democrat	M
18	Q182788	Newt Gingrich	86670	Republican	M
19	Q355522	Mitch McConnell	82061	Republican	M
20	Q11702	John Boehner	80793	Republican	M
21	Q380900	Chuck Schumer	75358	Democrat	M
22	Q314459	Harry Reid	73980	Democrat	M
23	Q324546	Marco Rubio	70349	Republican	M
24	Q434706	Elizabeth Warren	67699	Democrat	F
25	Q22212	Lindsey Graham	66895	Republican	M
26	Q439729	Rick Scott	65523	Republican	M
27	Q13133	Michelle Obama	65410	Democrat	F
28	Q2036942	Ted Cruz	64380	Republican	M
29	Q607	Michael Bloomberg	64366	Democrat	M
30	Q553254	Scott Walker	64326	Republican	M
